# Lower Levels of TAZ Expression Associated with Post-Surgical Wound Healing Complications in Soft Tissue Sarcoma Patients Treated with Preoperative Radiation

**DOI:** 10.3390/biomedicines13020344

**Published:** 2025-02-03

**Authors:** Jacob D. Gylten, Jane E. Persons, Benjamin J. Miller, Qiang An, Munir R. Tanas, Stephanie J. T. Chen

**Affiliations:** 1 Roy J. and Lucille A. Carver College of Medicine, The University of Iowa, Iowa City, IA 52242, USA; 2 Department of Pathology, The University of Iowa, Iowa City, IA 52242, USA; 3 Department of Orthopedic Surgery, The University of Iowa, Iowa City, IA 52242, USA

**Keywords:** sarcoma, preoperative radiation, wound healing, TAZ, Hippo pathway

## Abstract

**Background/Objectives:** Pre-operative radiation (Pre-RT) decreases local recurrence following soft tissue sarcoma (STS) resection but carries the risk of wound healing complications (WHCs). This study evaluated skin specimens and clinical characteristics of STS patients to (1) compare patients with and without Pre-RT, (2) compare Pre-RT patients with and without WHCs, and (3) explore associations between clinical characteristics and WHCs. **Methods:** This retrospective study included 54 adults who underwent STS resection with primary closure (Pre-RT n = 30). A pathologist who was blinded to the clinical outcomes evaluated the skin specimens microscopically. **Results:** Irradiated skin had lower vessel density and was more likely to lack hair follicles and sebaceous glands, consistent with the effects of radiation. Irradiated skin was also more likely to include plasma cells. Irradiated skin demonstrated higher mean TAZ H-scores; however, within the Pre-RT subset, those patients who developed WHCs demonstrated comparatively lower TAZ. **Conclusions:** This novel finding may suggest that higher TAZ in irradiated skin reflects a response to injury but that comparatively lower TAZ in irradiated skin might contribute to WHCs. Future studies should consider more focused evaluation of TAZ in STS resections with Pre-RT as they may help to predict WHCs when used in combination with other histologic factors and could suggest a therapeutic target.

## 1. Introduction

Soft tissue sarcoma (STS) includes a heterogeneous group of mesenchymal malignancies defined by the tissue of origin. STS frequently presents in the extremities as a slow-growing, painless mass [1]. Although STS accounts for only 1% of adult cancers [2], it is of concern due to its propensity for metastasis, local invasion, and recurrence. Due to its rarity and harmless progression, detection can often be delayed until patients present to specialty sarcoma centers after the tumor has already become large and invasive. STS resection morbidity, due to large incisions, postoperative dead space, thin skin-flaps, and resection of neighboring structures invaded by tumors, contributes to a high incidence of acute postoperative wound healing complications (WHCs), including infection, necrosis, dehiscence, and hematoma [3,4]. Acute WHCs represent a significant burden on the healthcare system and can require more intensive wound care, repeat surgeries, and even hospital admissions in some cases.

Previously, amputation was the mainstay of extremity STS management; today, limbs can often be salvaged due to advances in systemic therapy and, primarily, radiation. The addition of radiation is associated with improved long-term survival and decreased tumor recurrence in high-grade STS [5]. The ideal timing of preoperative or postoperative radiation therapy has been somewhat controversial [6]. Preoperative radiation therapy (Pre-RT) is the preferred radiation supplement to STS resection in the US [7]. It improves the chance of achieving negative resection margins by inducing tumor necrosis (particularly at the tumor edges) and enables the administration of lower doses of radiation, which limits exposure of healthy tissue and decreases the long-term side effects of radiation when compared to postoperative delivery.

While limb-salvage surgery with either Pre-RT or Post-RT results in improved survival rates compared to amputation or limb-sparing surgery alone [8], considerable side effects and postoperative complications remain with perioperative radiation treatment. One third of patients undergoing Pre-RT develop acute WHCs, nearly double the rate of patients treated with Post-RT [9]. In contrast, Post-RT patients are more likely to experience chronic complications, including joint fibrosis, immobility, fracture, and edema [10]. The decision between Pre-RT and Post-RT is highly nuanced, requiring consideration of multiple patient characteristics, including patient age, tumor location, tumor size, and patient comorbidities. Although acute WHCs following Pre-RT are treatable and more likely to resolve than the chronic complications that accompany Post-RT, acute WHCs are a frequent burden and thus an important focus of investigation.

In STS, patients treated with Pre-RT tend to develop WHCs associated with diabetes, tumor size, proximity to skin surface, lower-extremity tumor location, increased age, and higher BMI [11,12], which are mostly unavoidable factors. Multiple studies have attempted to identify and address modifiable risk factors to prevent WHCs for STS patients. These include prophylactic antibiotic administration [13], the use of vacuum-assisted closure devices [14], measuring the transcutaneous oxygen levels during the Pre-RT to surgery interval to guide the timing of surgical intervention [15], and adjustments of the NCCN-preferred 3- to 6-week interval between Pre-RT and surgery [7,16,17]. Although these efforts have been valuable, investigations into approaches to prevent WHCs for STS patients have overall been limited, too varied among studies, or yet to translate clinically.

Skin changes following radiation are an important focal point in the STS population treated with Pre-RT because of the potential implications for wound healing. In general, cutaneous repair and healing are attained through the complex and continuous steps of hemostasis, inflammation, proliferation, and remodeling [18,19]. WHCs result when these steps malfunction or fail to transition smoothly. While radiation-induced cutaneous changes including dermatitis, fibrosis, prolonged inflammation, and increased pro-inflammatory mediators are expected obstacles to healing in STS patients, the body of orthopedic and sarcoma literature contains few studies on derangements in these processes at the cellular level [20,21]. It is reasonable to conclude that more detailed analysis of the skin itself and the changes it undergoes following Pre-RT could highlight underlying pathologic processes in wound healing, in turn offering insight into the efforts to decrease WHCs.

**Figure 1:**
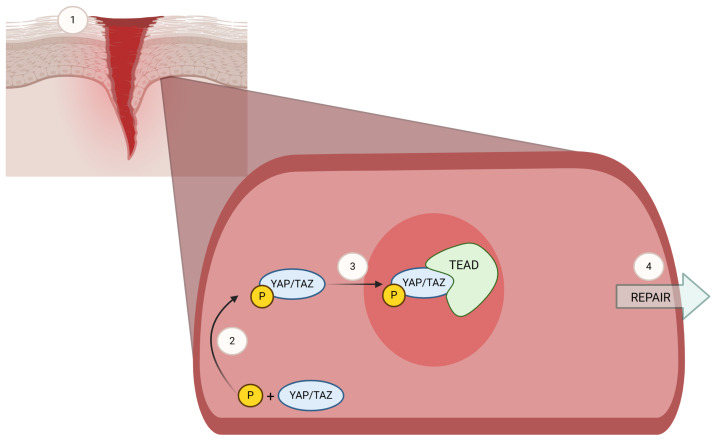
**YAP/TAZ in tissue injury and repair.** An illustration of activation of Yes-associated protein (YAP) and its paralog, transcriptional coactivator with PDZ-binding motif (TAZ), one of the final steps in the complex and only partially understood Hippo pathway [27,31]. Following cutaneous injury (1), YAP/TAZ undergoes activating phosphorylation events (2) and localizes to the nucleus of epithelial basal cells and migrating fibroblasts, where they combine with TEA domain (TEAD) family of transcription factors (3) to regulate wound healing (4). Created via https://BioRender.com.

A molecular marker of recent interest in both wound healing and STS is the transcriptional coactivator with PDZ-binding motif (TAZ). TAZ and its paralog Yes-associated-protein (YAP) independently combine with the TEAD (TEA domain) family of transcription factors to function as transcriptional regulators of the cell cycle. This is one of the final steps in the complex and only partially understood Hippo pathway, which is responsible for regulating cellular proliferation and tissue regeneration [22]. Walko et al. demonstrated nuclear localization of YAP–TEAD at the leading edge of migrating fibroblasts and basal cells of the epidermis during wound healing [23]. TAZ migration from the cytoplasm of non-injured tissue to the nucleus of injured tissue (Figure 1) is apparent in the epidermis, dermis, and hair follicles [22]. While the role of TAZ has been studied in normal tissue homeostasis, growth, and cutaneous wound healing, its behavior following radiation in STS patients is not yet fully understood. Moreover, YAP and TAZ activation have also been demonstrated in sarcomas [24,25,26,27]. Nuclear staining (activation) has been demonstrated in various sarcomas for both TAZ (33–66% of examined sarcomas) and YAP (50–53% of examined sarcomas) [24,28]. The activation of TAZ and YAP in these diseases has made these proteins an exploitable therapeutic target, with prior studies demonstrating efficacy in pharmacological inhibition of this pathway [26,29,30]. Thus, in STS patients, YAP and TAZ have multiple conflicting roles, being involved in oncogenesis, wound healing, and therapeutics.

To our knowledge, histologic examination and TAZ immunohistochemical analysis of skin following Pre-RT have yet to be evaluated in the context of WHCs following STS resection. Studying these features could provide an avenue to predict WHCs in this patient population, highlight the underlying processes of wound healing disorders, and potentially generate new opportunities for intervention.

The purpose of this study was to evaluate the histologic and clinical characteristics in patients who underwent surgical treatment for STS. First, the effects of Pre-RT were assessed by comparing the histologic and immunohistochemical findings in skin from patients with and without Pre-RT. Second, within the Pre-RT cohort, the histologic and immunohistochemical findings were compared between those patients who did and did not develop WHCs. Third, associations between the WHCs and clinical characteristics related to the patient, tumor, and treatment modality were explored.

## 2. Materials and Methods

This University of Iowa Institutional Review Board (IRB # 201403755)-approved retrospective study includes 54 adult patients with STS following limb-salvage resection with primary closure. The exposed group was defined as patients who received Pre-RT (n = 30) and the comparison group was defined as patients who did not receive Pre-RT prior to resection (n = 24).

### 2.1. Selection Criteria

**Figure 2:**
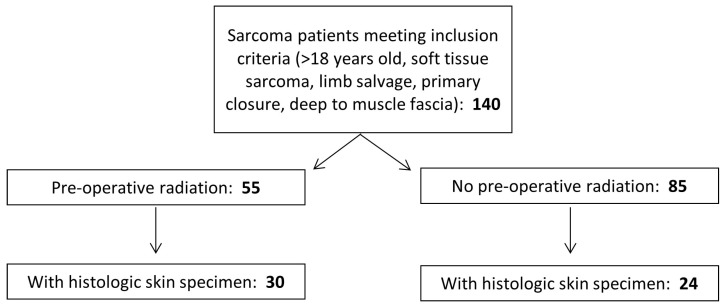
**Selection criteria for study cohort.** An initial cohort of 140 patients with soft tissue sarcoma met the inclusion criteria. Of these individuals, 55 received preoperative radiation therapy and were selected as the exposure group. The remaining 85 individuals were selected as comparison subjects. Pathology reports were reviewed to identify patients with archived formalin-fixed paraffin-embedded skin specimens available for analysis. Patients were excluded if their surgical specimens did not include full-thickness skin, or if the archived tissue was exhausted or was not available. The final cohort included 54 patients (31 with preoperative radiation therapy and 24 without).

Patients were identified from a pool of 140 patients who underwent resection of pelvic or extremity STS at the University of Iowa Hospitals and Clinics (UIHC) from 2011 to 2019 and met inclusion criteria (>18 years old, primary STS, limb-salvage resection, primary closure, and tumor deep to muscle fascia) (Figure 2). Tissue was obtained through the usual course of diagnosis and treatment.

### 2.2. Histologic Analysis

When available, archived hematoxylin and eosin (H&E)-stained slides from surgical resection specimens were reviewed for patients meeting initial inclusion criteria. These 4-micron-thick tissue sections were cut from formalin-fixed paraffin-embedded (FFPE) tissue blocks prepared using standard protocols in the UIHC clinical pathology laboratory during routine diagnosis. Skin was included with surgical resection specimens from 61 of the initial 140 patients who met inclusion criteria. Full-thickness skin specimens adequate for study inclusion were those possessing a complete epidermis, dermis, and at least a focally identifiable hypodermis (to ensure proper assessment of dermal thickness). Skin with biopsy site changes or scar was excluded from analysis. When H&E-stained slides were unavailable, archived FFPE tissue blocks were used to prepare 4-micron-thick H&E-stained sections in the Histology Research Laboratory (HRL) at the UIHC using standard protocols. Of the 61 resections with skin, FFPE tissue blocks were available for 54 patients, who comprise the final study sample.

Histopathologic examination was performed by a board-certified pathologist with fellowship training in dermatopathology, who was blinded to all clinical parameters. Tissue specimens were histologically evaluated for inflammation, dermal thickness, vessel density, elastin organization, and dermal fibroblast expression of TAZ.

Inflammation, dermal thickness, and vessel density were evaluated using H&E-stained slides. Inflammation was assessed via multiple modalities: location, percent involvement, cell type, and cell count. Location of the inflammation was captured as perivascular, periadnexal, interstitial, intraepidermal, and/or interface. Percent involvement was defined as the estimated percent of the total epidermal and dermal area involved regarding inflammation. Cell type was noted as the presence or absence of lymphocytes, histiocytes, neutrophils, plasma cells, eosinophils, and multinucleated giant cells. Inflammatory cell count was defined as the number of inflammatory cells within the most cellular field at 400× magnification. Representative dermal thickness was measured microscopically to the nearest 0.5 mm, from the dermal–epidermal junction to the superficial-most aspect of the hypodermis (adipose). Vessel density was captured as the number of vessels in cross-section per ten random fields at 400× magnification.

Elastin fiber organization was evaluated using elastic trichrome staining. Elastic trichrome stains for each specimen were prepared in the HRL using standard protocols and FFPE tissue blocks. Elastin fiber organization was evaluated as previously described by Kung et al., who used the Verhoeff–van Gieson stain (a comparable elastic tissue fiber stain) to evaluate skin in a cohort of patients treated with post-mastectomy radiation [32]. These authors described scoring elastin fiber disorganization on a scale of 0 to 3 as follows: 0 for normal elastin fiber organization, 1 for mild disorganization (>0 to ≤25% disorganized), 2 for moderate disorganization (>25 to ≤50% disorganized), and 3 for severe disorganization (>50% disorganized).

### 2.3. Immunohistochemical Staining

**Figure 3:**
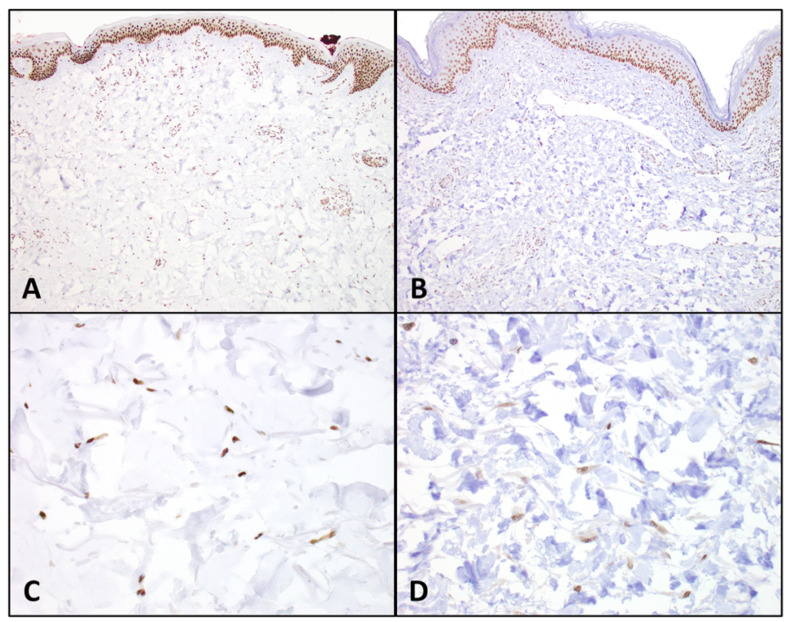
**TAZ immunohistochemistry in dermal fibroblasts.** Comparison of selected high and low TAZ expression by immunohistochemistry evaluated on light microscopy using an Olympus BX43 microscope. Panels (**A**,**C**) show a case with one of the highest H-scores (score = 300) for TAZ expression, with strong nuclear staining noted in virtually all dermal fibroblasts [100× and 400× magnification, respectively]. Panels (**B**,**D**) show a case with one of the lower H-scores (score = 120) for TAZ expression, with weak and patchy TAZ expression in dermal fibroblasts [100× and 400× magnification, respectively].

Immunohistochemical staining for TAZ was also performed for each specimen using FFPE tissue blocks and anti-TAZ antibody (mouse monoclonal 1H9; catalog #LS-C173295; 1:50 primary dilution) obtained from LifeSpan BioSciences (Seattle, WA, USA). Dermal fibroblast nuclei were evaluated for TAZ expression using endothelial cells in dermal blood vessels as an internal control. Staining intensity was classified as 3+ (strong, equivalent to endothelial cell intensity), 2+ (moderate), or 1+ (weak) (Figure 3). The percentages of fibroblast staining at primary and secondary intensities were recorded. TAZ expression was quantified using H-scores (intensity * percentages of fibroblast staining at that intensity). H-scores were determined for the primary and secondary staining intensities. The sum of these primary and secondary H-scores represented the total H-score for each patient. 

### 2.4. Clinical Data Abstraction

Lastly, clinical characteristics relating to patient (age and sex), tumor (size, grade, and location), and treatment (radiation and neoadjuvant chemotherapy) were evaluated in relation to WHCs. A WHC was defined as any skin condition requiring an invasive secondary surgery, readmission, or complex wound management directly related to the primary resection. This included infection, necrosis, hematoma, and prolonged dressing changes occurring within 4 months of the primary surgery. Skin grafts, but not rotational muscle flaps, were an exclusion criterion as to isolate native skin healing after surgery.

### 2.5. Statistical Analysis

Comparisons were performed using chi-squared and Fisher’s Exact tests for categorical bivariate measures of association and Wilcoxon Rank Sum for continuous variables in group comparison. In primary models, logistic regression analysis was used to (1) compare patients with and without Pre-RT, (2) compare patients with and without WHCs within the subset of patients who received Pre-RT, and (3) explore associations between clinical characteristics and the development of WHCs. Haldane–Anscombe correction was used for calculating odds ratios. Significance was set at the alpha = 0.05 level. All statistical analyses were conducted using SAS 9.4 (SAS Institute Inc., Cary, NC, USA).

## 3. Results

The first aim was to compare patients with and without Pre-RT (Table 1). The mean TAZ H-scores were significantly higher among the patients with Pre-RT (276.5 ± 38.8 vs. 253.9 ± 48.5; *p* = 0.017). In addition, plasma cells were more frequently present in radiated skin than non-radiated skin (*p* = 0.026). Radiated skin was also more likely to have an absence of hair follicles (*p* = 0.015) and sebaceous glands (*p* = 0.016), and to demonstrate lower vessel density (*p* = 0.041). Logistic regression was performed to calculate the odds of these statistically significant features occurring in patients with and without Pre-RT. Those patients who received Pre-RT had increased odds of having a skin specimen that included plasma cells (OR 28.9, 95% CI 1.5–553.4), lacked sebaceous glands (OR 11.9, 95% CI 1.4–105.3), lacked hair follicles (OR 4.3, 95% CI 1.1–31.0), and had lower blood vessel density (OR 5.8, 95% CI 1.3–14.7).

The second aim was to compare patients with and without WHCs within the subset of patients who received Pre-RT (Table 2). Those patients with WHCs had significantly lower mean TAZ H-scores than the patients without WHCs (260.0 ± 57.3 versus 282.5 ± 29.0; *p* = 0.040). The Pre-RT patients with WHCs also trended toward more organized elastin, more frequent presence of neutrophils and hair follicles, and lower concentrations of inflammatory cells; however, none of these findings were statistically significant.

The third aim was to explore potential associations between the clinical characteristics and WHCs. The clinical characteristics of patient, tumor, and treatment details were not significantly associated with WHCs within the full cohort (Table 3) or within the subset of patients who received Pre-RT (Table 4).

## 4. Discussion

The primary goal of this study was to assess an extensive panel of histologic characteristics and TAZ immunohistochemical expression in STS patient skin treated with Pre-RT given their predisposition toward WHCs. The results demonstrate an association between Pre-RT and the absence of hair follicles and sebaceous glands, decreased vessel density, and presence of cutaneous plasma cells. The adnexal and vascular changes are consistent with what would be expected following radiation. The most novel findings, however, center around TAZ expression.

The current study demonstrates increased TAZ expression in irradiated skin overall, with lower levels of TAZ expression in irradiated skin associated with the development of WHCs. Previous animal models and human cell cultures display TAZ nuclear localization in early cutaneous wound healing and suggest that loss of TAZ may impair wound healing [22,31]. The removal of YAP and TAZ via siRNA interference in mice with full-thickness dermal wounds resulted in lowered expression of TGF-beta-1 signaling pathway components, which implies that YAP and TAZ may modulate their expression and subsequently influence wound healing [22]. The modulation of TGF-beta is important for fibroblast migration, extracellular matrix deposition (e.g., elastin), epithelial healing, and the recruitment of inflammatory cells such as neutrophils and macrophages [33]. Radiation-induced fibrosis has also been shown to be mediated by TGF-beta-1 [34]. It seems that altered expression of TGF-beta-1 may interfere with wound healing since modulating the TAZ–TEAD complex has been shown to have an impact on its expression and wound healing. In the Pre-RT patient cohort, the increased TAZ expression may reflect a healing response to radiation injury. Furthermore, in the irradiated patients who later developed WHCs, the healing response may have been inadequate, as represented by their lower TAZ H-scores relative to those irradiated patients who did not develop WHCs.

TAZ is also involved in proper angiogenesis. Kim and colleagues showed that mouse endothelial cell knockdown of both YAP and TAZ together resulted in phenotypic endothelial cell alterations, disrupted barrier function, and delayed sprouting angiogenesis. The knockdown of YAP or TAZ alone in the retina showed much less affected endothelial cell development, suggesting somewhat redundant roles of YAP and TAZ in angiogenesis and vessel integrity [35]. In the current study, the patients with Pre-RT demonstrated both increased TAZ expression and decreased vascularity. It is possible that, in this group, any TAZ-associated increase in vascularity may have been obscured by accompanying decreases in vascularity that are characteristically expected in irradiated skin. The demonstration of higher TAZ H-scores among the patients with Pre-RT supports the reported importance of this protein in healing, but it alone may be inadequate for angiogenesis in the context of irradiated skin.

A trend toward increased neutrophil frequency was observed in those patients with WHCs, but it was not statistically significant. This supports a connection between prolonged inflammation and WHCs despite the lack of this trend in the overall inflammatory cell count. Although neutrophils are helpful in early wound healing, their persistence can contribute to non-healing wounds through continued release of reactive oxygen species, degradative enzymes, and pro-inflammatory mediators, which are risk factors for skin damage [18,36].

The decreased hair follicles and sebaceous glands in the skin of irradiated patients in this study are consistent with previous descriptions of irradiated skin and radiation dermatitis [37]. Keratinocytes from the pilosebaceous units, along with eccrine glands, contribute to re-epithelialization in skin wounds [18,38], but their decrease in the irradiated patients of this study did not translate to an association with more WHCs. This may suggest that factors other than a reduced capacity for re-epithelialization play a significant role in WHCs.

Two findings in the current study appear to be contrary to the limited applicable published literature. First, the skin samples in the patients who received Pre-RT did not demonstrate greater elastin disorganization than those who had no Pre-RT, in contrast to what has been reported previously by Kung et al. However, those authors reported peak elastin fiber disorganization at around 4 months [32]. The shorter interval of less than 6 weeks from the last radiation dose to surgery in the current study may not have allowed time for elastin fiber disorganization to completely develop and reproduce this finding. Further, the results of this study of patients with breast radiation may not be generalizable to skin of other anatomic sites. Secondly, although an irradiated mouse model showed significantly increased dermal thickness at 4 and 12 weeks post-irradiation when compared with the controls [37], the dermal thickness did not differ between the irradiated and non-irradiated patients in the current study. We also observed no difference in dermal thickness between irradiated patients with or without WHCs.

One difficult-to-explain finding is that the skin samples in those patients who received Pre-RT were more likely to contain plasma cells than the skin samples in patients who did not receive radiation. To our knowledge, plasma cells have not been explicitly described in cutaneous infiltrates following radiation. However, a study of human and murine skin demonstrated the development of a plasma-cell-supportive microenvironment and the resultant accumulation of a large number of plasma cells secreting IgM in response to chronic inflammation [39]. It is also well known that plasma cells are more prevalent at certain anatomic sites, particularly the head and neck and anogenital regions [40]. These may represent possible explanations for this finding, but its overall significance is uncertain.

The clinical characteristics in this study had no significant associations with WHCs. This is likely due to the relatively low numbers and heterogeneity of the sample population, a common limitation in studies investigating sarcoma, as the observed factors (e.g., age and tumor location) have shown significance in larger studies [11]. Despite observing no WHCs in those patients with an upper extremity tumor location or tumor size of less than 5 cm, the small cohort size limits the statistical power behind those findings. Although the sample size is a limitation of this study, an important strength is that it was conducted at the University of Iowa Hospitals and Clinics, one of only several sarcoma centers in the US. Future studies may consider recruiting at the national level to increase their sample size.

Given the importance of TAZ in wound healing, it is interesting that cases with significantly lower TAZ H-scores experienced WHCs. TAZ’s location downstream of several signal transduction pathways, conserved expression in various tissues, and interactions with other cellular mediators are factors that contribute to its importance in skin healing. An additional consideration is that interventions targeting TAZ with the intent of limiting tumor growth or metastasis may have an unintended consequence of wound healing compromise. Although the TAZ H-score differences in this cohort are not discriminative enough to be a stand-alone predictor of WHCs, future studies should conduct a more focused evaluation of TAZ expression levels in STS resections with Pre-RT as they may help to predict WHCs when used in combination with other histologic factors and could suggest a therapeutic target.

## Figures and Tables

**Table 1:** **Comparison of microscopic findings between patients with and without preoperative radiation therapy (Pre-RT)** (n = 54).

	Pre-RT (n = 30)	No Pre-RT (n = 24)	*p*-Value *
**Elastin Organization** ^†^			
0–1	23 (76.7%)	15 (62.5%)	0.257
3–4	7 (23.3%)	9 (37.5%)	
**Neutrophils** ^‡^	7 (23.3%)	1 (4.2%)	0.0633
**Plasma Cells** ^‡^	11 (36.7%)	0 (0%)	**0.0255**
**Inflammatory Cells** ^§^			
>100/HPF	13 (43.3%)	8 (33.3%)	0.4538
≤100/HPF	17 (56.7%)	16 (66.7%)	
**Hair Follicles** ^‡^	14 (46.7%)	19 (79.2%)	**0.0149**
**Eccrine Glands** ^‡^	30 (100%)	23 (95.8%)	0.4444
**Sebaceous Glands ^‡^**	1 (3.3%)	7 (29.2%)	**0.0163**
**Dermal Thickness (mm)**			
≥4	6	7	0.4337
<4	24	17	
**Vessels/10 HPF ^§^**			
>30	2	7	**0.0414**
≤30	28	17	
**Total TAZ H-score**^¶^ Mean ± SD	276.5 ± 38.8	253.9 ± 48.5	**0.017**

* **Bolded text** indicates statistical significance at the alpha = 0.05 level. ^†^ Elastin organization was quantified using an ordinal scale with the following values: 0 = normal organization; 1 = mild (>0% to ≤25%) disorganization; 2 = moderate (>25% to ≤50%) disorganization; 3 = severe (>50%) disorganization. ^‡^ Reported as the number (%) of patients with at least one representative of the specified histologic entity present in the examined skin specimen. ^§^ HPF = high-power field. For the purposes of this study, HPF is defined as 400× magnification. ^¶^ H-score was used to quantify TAZ expression and was calculated as (staining intensity) * (% of fibroblast staining at that intensity). Staining intensity was classified as 3+ (strong, equivalent to endothelial cell staining), 2+ (moderate), or 1+ (weak). The percentages of fibroblast staining at primary and secondary intensities were summed to produce the total H-score.

**Table 2:** **Comparison of microscopic findings in patients treated with preoperative radiation with and without wound healing complications (WHCs)** (n = 30).

	WHC (n = 8)	No WHC (n = 22)	*p*-Value *
**Elastin Organization** ^†^			
0–1	8 (100%)	15 (68.2%)	0.1434
3–4	0 (0%)	7 (31.8%)	
**Neutrophils** ^‡^	4 (50.0%)	3 (13.6%)	0.0596
**Plasma Cells** ^‡^	3 (37.5%)	8 (36.4%)	1.0000
**Inflammatory Cells** ^§^			
>100/HPF	2 (25.0%)	11 (50.0%)	0.4069
≤100/HPF	6 (75.0%)	11 (50.0%)	
**Hair Follicles** ^‡^	5 (62.5%)	9 (40.9%)	0.4171
**Eccrine Glands** ^‡^	8 (100%)	22 (100%)	–
**Sebaceous Glands ^‡^**	1 (12.5%)	0 (0%)	0.2667
**Dermal Thickness (mm)**			
≥4	2 (25.0%)	4 (18.2%)	0.6452
< 4	6 (75.0%)	18 (81.8%)	
**Vessels/10 HPF ^§^**			
>30	1 (12.5%)	1 (4.5%)	0.4690
≤30	7 (87.5%)	21 (95.5%)	
**Total TAZ H-score**^¶^ Mean ± SD	260 ± 57.32	282.5 ± 29.02	**0.0402**

* **Bolded text** indicates statistical significance at the alpha = 0.05 level. ^†^ Elastin organization was quantified using an ordinal scale with the following values: 0 = normal organization; 1 = mild (>0% to ≤25%) disorganization; 2 = moderate (>25% to ≤50%) disorganization; 3 = severe (>50%) disorganization. One tissue block was not available for elastin staining in WHC group. ^‡^ Reported as the number (%) of patients with at least one representative of the specified histologic entity present in the examined skin specimen. ^§^ HPF = high-power field. For the purposes of this study, HPF is defined as 400× magnification. ^¶^ H-score was used to quantify TAZ expression and was calculated as (staining intensity) * (% of fibroblast staining at that intensity). Staining intensity was classified as 3+ (strong, equivalent to endothelial cell staining), 2+ (moderate), or 1+ (weak). The percentages of fibroblast staining at primary and secondary intensities were summed to produce the total H-score.

**Table 3:** **Patient, tumor, and treatment characteristics** (n = 54).

Patient Characteristics	Total N (%)	WHC (n = 11)	No WHC (n = 43)	*p*-Value *
**Sex**				
Male	30 (55.6%)	5 (45.5%)	25 (58.1%)	0.5104
Female	24 (44.4%)	6 (54.5%)	18 (41.9%)	
**Age**				
**Tumor Characteristics**				
<65	26 (48.1%)	5 (45.5%)	21 (48.8%)	0.8412
≥65	28 (51.9%)	6 (54.5%)	22 (51.2%)	
**Size (cm)**				
<5	4 (7.4%)	0 (0%)	4 (9.3%)	0.3517
5–10	17 (31.5%)	2 (18.1%)	15 (34.9%)	
>10	33 (61.1%)	9 (81.8%)	24 (55.8%)	
**Grade**				
Low	6 (11.1%)	2 (18.1%)	4 (9.3%)	0.7075
Intermediate	24 (44.4%)	4 (36.4%)	20 (46.5%)	
High	24 (44.4%)	5 (45.5%)	19 (44.2%)	
**Location**				
Upper Extremity	11 (20.4%)	0 (0%)	11 (25.6%)	0.0958
Lower Extremity	41 (75.9%)	10 (90.9%)	31 (72.1%)	
Axial	2 (3.7%)	1 (9.1%)	1 (2.3%)	
**Treatment Characteristics**				
**Radiation**				
Preoperative	30 (55.6%)	8 (72.7%)	22 (51.2%)	0.6956
Postoperative	12 (22.2%)	2 (18.2%)	10 (23.2%)	
**Neoadjuvant Chemotherapy**				
Cytotoxic	9 (16.7%)	1 (9.1%)	8 (18.6%)	0.7334
Immunotherapy	1 (1.9%)	0 (0%)	1 (2.3%)	
None	44 (81.5%)	10 (90.9%)	34 (79.1%)	

* **Bolded text** indicates statistical significance at the alpha = 0.05 level.

**Table 4:** **Clinical characteristic comparison of wound healing complications in patients who received preoperative radiation therapy (Pre-RT)** (n = 30).

	WHC (n = 8)	No WHC (n = 22)	*p*-Value *
**Sex**			
Male	4	14	0.6779
Female	4	8	
**Age**			
<65	5	12	1.0000
≥65	3	10	
**Tumor Size (cm)**			
<5	0	1	1.0000
5–10	2	7	
≥10	6	14	
**Tumor Grade**			
Low	1	2	1.0000
Intermediate	4	13	
High	3	7	
**Location**			
Upper Extremity	0	6	0.2560
Lower Extremity	7	15	
Axial	1	1	
**Neoadjuvant Chemotherapy**			
Cytotoxic	0	4	0.6715
Not Cytotoxic	8	18	

* **Bolded text** indicates statistical significance at the alpha = 0.05 level.

## Data Availability

The original contributions presented in this study are included in the article. Further inquiries can be directed to the corresponding author.
